# Effects of cIAP-1, cIAP-2 and XIAP triple knockdown on prostate cancer cell susceptibility to apoptosis, cell survival and proliferation

**DOI:** 10.1186/1476-4598-8-39

**Published:** 2009-06-23

**Authors:** Catherine Gill, Catherine Dowling, Amanda J O'Neill, R William G Watson

**Affiliations:** 1 UCD School of Medicine and Medical Science, University College Dublin, Dublin, Ireland; 2 UCD Conway Institute of Biomolecular and Biomedical Research, University College Dublin, Dublin, Ireland

## Abstract

**Background:**

Manipulating apoptotic resistance represents an important strategy for the treatment of hormone refractory prostate cancer. We hypothesised that the Inhibitor of Apoptosis (IAP) Proteins may be mediating this resistance and knockdown of cIAP-1, cIAP-2 and XIAP would increase sensitivity to apoptosis.

**Methods:**

cIAP-1, cIAP-2 and XIAP where knocked down either individually or in combination using siRNA in androgen independent prostate cancer PC-3 cells as confirmed by real-time PCR and western blotting. Cells were then treated with TRAIL, Etoposide, or Tunicamycin, and apoptosis assessed by PI DNA staining. Apoptosis was confirmed with Annexin V labelling and measurement of PARP cleavage, and was inhibited using the pan-caspase inhibitor, zVAD.fmk. Clonogenic assays and assessment of ID-1 expression by western blotting were used to measure recovery and proliferation.

**Results:**

PC-3 are resistant to TRAIL induced apoptosis and have elevated expression of cIAP-1, cIAP-2 and XIAP. Combined knockdown sensitised PC-3 to TRAIL induced apoptosis, but not to Etoposide or Tunicmycin, with corresponding increases in caspase activity and PARP cleavage which was inhibited by ZVAD.fmk. Triple knock down decreased proliferation which was confirmed by decreased ID-1 expression.

**Conclusion:**

Simultaneous knock down of the IAPs not only sensitised the PC-3 to TRAIL but also inhibited their proliferation rates and clonogenic survival. The inability to alter sensitivity to other triggers of apoptosis suggests that this effect is specific for death receptor pathways and knock down might facilitate immune-surveillance mechanisms to counter cancer progression and, in combination with therapeutic approaches using TRAIL, could represent an important treatment strategy.

## Background

Prostate cancer is the most common male cancer in the western world and the second leading cause of death amongst men. In its initial stages, when confined to the prostate gland, the disease is treatable and even curable. However, prostate cancer is often diagnosed when it is no longer organ confined, and although this condition is also initially amenable to treatment, eventually an androgen independent cancer develops, for which there is currently no cure.

One of the difficulties in treating prostate cancer is that cells become highly resistant to cell death. Thus although depriving an androgen-sensitive tumour of androgen initially results in cell death by apoptosis, once the tumour is no longer dependent on androgen for survival, this treatment becomes ineffective [1]. This resistance is also associated with resistance to conventional chemotherapeutic agents. Consequently much of the focus of current research is on understanding the basis of altered apoptotic signalling in prostate cancer, in an effort to better understand and target cell death pathways for the treatment of this disease [2].

It is well established that anti-apoptotic proteins such as Bcl-2 [3] and Hsp27 [4] are over expressed in prostate cancer and contribute to the apoptosis resistant phenotype. In addition, the proteins of the Inhibitor of Apoptosis (IAP) Protein family are also over expressed and are thought to contribute to treatment resistance [5-8]. In prostate cancer, increased expression of cIAP-1, cIAP-2, XIAP and survivin have been found in biopsy specimens from all stages of the disease, suggesting an important role in development and progression [6]. In addition, altered expression of IAPs have been reported to contribute to both anti-androgen [8] and cisplatin [7] resistance in prostate cancer cell lines.

The pro-survival function of IAPs stems in part from their ability to bind to and inhibit the activity of caspases. This is certainly the case for XIAP which directly inhibits caspases -3, -7 and -9 [9]. While cIAP1 and cIAP2 can also bind caspases this does not always result in caspase inhibition in cell free systems [10], suggesting that IAPs other than XIAP are not truly inhibitors of caspases. While a number of reports would suggest that they do not inhibit caspases directly, there is a growing body of evidence that IAPs such as cIAP1 and cIAP2 may inhibit caspase activity and apoptosis in a more indirect manner particularly at the level of death receptor signalling [11-14].

Several studies have demonstrated that targeting individual IAPs leads to increased sensitivity of a variety of cancer types to apoptosis [15-19]. In prostate cancer cells, targeted inhibition of survivin increases sensitivity to flutamide [8] and paclitaxel [20]. XIAP inhibition was reported to increase sensitivity to cisplatin [21], while an antisense oligonucleotide targeting XIAP has shown promise in preclinical studies [22]. However, little is known about the effect of specifically targeting cIAP1 or cIAP2 in prostate cancer cells. In addition, while a number of studies have investigated the effects of individual IAP knockdown on cell survival [15-19], few studies have examined the effect of combined knockdown of two or more IAPs. This is particularly important given that increased expression of multiple IAPs has been observed in cancer biospecimens [5], suggesting that targeting an individual protein may be insufficient to alter a tumours sensitivity to cell death-inducing strategies *in vitro *or *in vivo*.

As well as their role in apoptosis inhibition, IAPs have a number of additional functions. These include a role in cell cycle regulation [23], differentiation [24], immunity [25], and ubiquitination and targeting of proteins for degradation [26]. In terms of a pro-survival function that does not rely on direct apoptosis inhibition, cIAP1 has recently been implicated as an oncogene whereby it cooperates with Yap 1 to promote tumourogenesis [27]. Thus IAPs may have other pro-survival effects in addition to direct apoptosis inhibition, and the effect of knockdown of single or multiple IAPs on cell survival merits further investigation.

In the current study we wished to determine whether IAP knockdown could sensitise prostate cancer cells to apoptosis, and to investigate the relative contribution of cIAP-1, cIAP-2 and XIAP to this process. As well as an effect on cell death, the impact of specific inhibition of single or multiple IAPs on long term survival of prostate cancer cells was investigated. Here we demonstrate that combined knockdown of either cIAP-1 and XIAP, or all three IAPs, was required to significantly enhance cytotoxicity in PC-3 cells. This effect was caspase-dependent and specific for TRAIL treatment. While IAP knockdown in the absence of additional treatment did not lead to a detectable increase in apoptosis, it significantly decreased long-term survival and proliferation, an effect which was associated with loss of the proliferation marker ID-1. This study therefore demonstrates the importance of targeting multiple IAPs to enhance sensitivity to apoptosis-inducing agents, and demonstrates that even in the absence of an apoptosis stimulus, tumour cell survival may be inhibited by targeting a specific combination of IAPs.

## Methods

### Cell culture and treatment

The human prostate cancer cell line PC-3 was purchased from the American Type Culture Collection (ATCC) and maintained in RPMI-1640 medium supplemented with 10% FBS, 50 U/ml penicillin/50 μg/ml streptomycin and 2 mM L-glutamine (Invitrogen). Twenty four hours post transfection, fresh media was added to cells before treatment with TRAIL (Peprotech), Etoposide, or Tunicamycin (Sigma). In caspase inhibition experiments zVAD.fmk (R&D Systems) was added for 1 hour prior to treatment with TRAIL.

### Caspase activity assays

Cells were harvested and pelleted by centrifugation at 200 × g for 5 min, resuspended in ice-cold PBS and centrifuged for a further 5 min at 200 × g. The pellet was then resuspended in a 25 μl volume of PBS, transferred to a microtitre plate and snap-frozen. Assay buffer (100 mM HEPES, 10% sucrose, 0.1% CHAPS, 5 mM DTT, 0.0001% NP-40, pH 7.25) containing 50 μM substrate (DEVD-AMC, Peptide Institute Inc., Japan) was added to samples. Cleavage of the fluorogenic substrate was measured using a Wallac 1420 Multilabel counter (PerkinElmer life sciences) with 355 nm excitation and 460 nm emission wavelengths. Protein concentration of samples was determined by the Bradford method and activity was expressed as change in fluorescence/min/mg of protein.

### Western blot analysis

Cells were harvested, washed once in PBS and lysed in whole cell lysis buffer (20 mM Hepes pH 7.5, 350 mM NaCl, 1 mM MgCl_2_, 0.5 mM EDTA, 0.1 mM EGTA, 1% NP-40, 1 mM NaF and 1 mM Na_3_VO_4, _plus cocktail of protease inhibitors). For PARP westerns cells were lysed in Urea lysis buffer (7 M Urea, 2 M Thiourea, 2% CHAPS, 1% DTT, 0.8% Pharmalyte, protease inhibitors). Total cellular protein was determined by means of the Bradford method. Equal amounts of protein (20–30 μg) were subjected to SDS polyacrylamide gel electrophoresis on 8–12% gels before being transblotted onto Immobilin P (Millipore) membranes. Western blotting was performed using antibodies to cIAP-1, cIAP-2, XIAP (R&D Systems), caspase-3, caspase-8, caspase-9 (Cell Signalling Technology), PARP (Biomol), ID-1 (Santa Cruz), β-Actin (Sigma) and GAPDH (Chemicon), followed by incubation with the appropriate horseradish peroxidase-conjugated secondary antibodies. Signals were detected using ECL™ (Amersham Biosciences).

### Quantification of Apoptosis and Viability

Apoptotic events were described as a percentage of total events with hypodiploid DNA assessed by propidium iodide incorporation. Cells were harvested by trypsinisation, permeabilised with a hypotonic fluorochrome solution (50 mg/ml PI, 3.4 mM sodium citrate, 1 mM Tris, 0.1 mM EDTA, and 0.1% Triton X-100) and incubated on ice for 10 min prior to analysis. Samples were run on an Epics XL-MCL Coulter Elite Cytometer (Coulter Cytometry). Five thousand events were gated on PI intensity and analysed using Mplus software.

### Real Time PCR

Total RNA was extracted using Trizol reagent (Invitrogen) using standard procedures. Reverse transcription was carried out with 1 μg total RNA using random primers and SUPERSCRIPT II (Invitrogen). Real-Time PCR TaqMan assay was used to quantitate the relative gene expression levels of cIAP1, cIAP2 and XIAP, with ribosomal 18S gene used as an endogenous control for normalisation of target genes. Primers and probes supplied as pre-developed assay, were combined with Taqman Universal PCR Master Mix (Applied Biosystems) and cDNA. Amplification was performed on the ABI 7900 HT Sequence Detection System at default thermal cycling conditions: 2 min at 50°C, 10 min at 95°C, 40 cycles of 15 sec at 95°C, and 1 min at 60°C. Results were analysed using the standard curve method of analysis.

### RNA interference

siRNAs targeting cIAP-1, cIAP-2, XIAP, and Silencer Negative Control siRNAs were purchased from Ambion. PC-3 cells were seeded in antibiotic-free media for 24 h prior to transfection with 0.5 nM siRNA targeting cIAP-1, cIAP-2, XIAP, or combinations thereof, and corresponding non-targeting controls. A total of 1.5 nM of siRNA was added to each experiment made up of the target siRNA and topped up with the appropriate concentration of non-targeted controls where appropriate. Transfections were carried out in OptiMem medium (Gibco) using Lipofectamine 2000 transfection reagent (Invitrogen). Twenty four hours post transfection cells were harvested and assayed for RNA and protein expression levels of the target of interest. At the same time corresponding samples were treated as described in the text.

### Annexin V staining and flow cytometry

Cells were harvested by trypsinisation, washed in cold PBS, and resuspended in 100 μl of Annexin V/calcium binding buffer solution (IQProducts). Following incubation at RT for 15 min in the dark, samples were placed on ice and 300 μl of calcium binding buffer and 400 μl of PI-viability solution were added. Samples were run on an Epics XL-MCL Coulter Elite Cytometer (Coulter Cytometry). A minimum of 104 events were acquired and analysed using Mplus software.

### Crystal violet assay

Following transfection, cells were grown for up to 5 days in 6 well plates. To determine cell number at each timepoint, cells were fixed in 1% glutaraldehyde solution, washed, incubated with 1% crystal violet (Sigma-Aldrich, UK), washed, solubilised with 1% Triton X-100, and analysed at an absorbance of 590 nm (Tecan UK Ltd., UK) as previously described [28].

### Clonogenic survival assay

Twenty four hours post-transfection cells were trypsinised, counted, and accurately seeded at densities of 1000 per well in 6-well plates. Cells were grown for 2 weeks until colonies were of sufficient size for staining and counting. Colonies were washed in PBS, stained with fixation-staining solution (glutaraldehyde 6% (v/v), crystal violet 0.5% (w/v) in H_2_O), washed by immersing in tap water and then air dried at room temperature. Colonies were counted to determine surviving fraction using the following formulae:





### Statistical analysis

Statistical analysis was carried out using unpaired student t-test (Graphpad InStat 3 software, GraphPad Software, Inc., San Diego). Results were considered statistically significant where p < 0.01, and results are expressed as mean ± the standard deviation.

## Results

### TRAIL induced caspase activation in PC-3 cells does not lead to apoptosis

**Figure 1:**
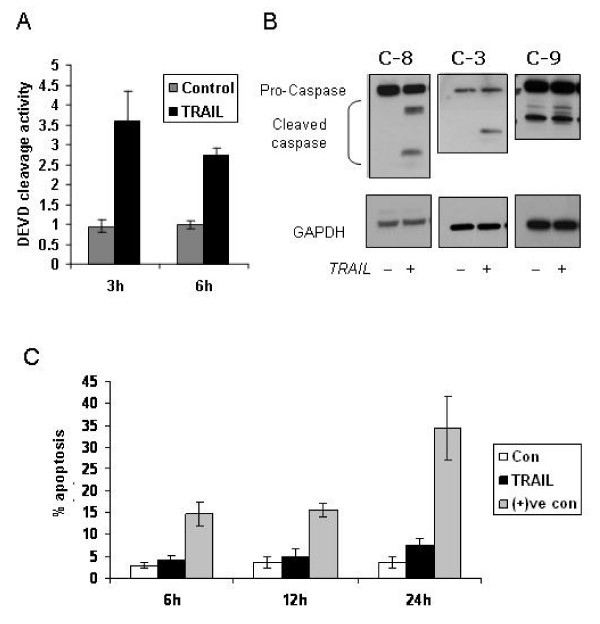
**TRAIL induced caspase activation in PC-3 cells does not lead to apoptosis**. **A**. PC-3 cells were treated with TRAIL (25 ng/ml) for the indicated times. Caspase (DEVDase) activity was measured using a fluorometric assay as described in materials and methods with activity relative to control cells. **B**. Cells were treated with TRAIL (25 ng/ml) for 3 hours. The expression levels of caspases -3, -8, and -9, and their cleaved products were detected by Western blotting. Data shown is representative of a minimum of three independent experiments. **C**. Apoptosis (%) was assessed by propidium iodide DNA staining and flow cytometry after 6 – 24 hours of TRAIL treatment in PC-3 cells. Treatment of TRAIL in combination with resveratrol was included at all time points as a positive control ((+)ve con) for apoptosis induction.

Androgen independent prostate cancer cell lines are highly resistant to apoptosis inducing agents such as TRAIL [29]. It is thought that this is due in part to altered expression of apoptosis regulatory molecules which impairs apoptotic signalling. In order to investigate at which point resistance to TRAIL is occurring, PC-3 cells were treated with TRAIL and caspase activity was measured at 3 and 6 hours post-treatment. As shown in Figure 1, TRAIL treatment leads to a significant induction of caspase activity as assessed by DEVD cleavage (Figure 1A), and the expression of a cleaved caspases-3, -8 and -9 protein as early as 3 hours (Figure 1B), however, this did not result in a corresponding increase in cell death even at 24 hours (Figure 1C). Thus although TRAIL can initiate an apoptotic signal in PC-3 cells, there is a block present which prevents apoptosis going to completion. Activation of caspases in the absence of cell death suggests that a protein or family of proteins may be over expressed in these cells which inhibits the apoptosis pathway. IAPs are known to inhibit apoptosis at the level of caspases, not only inhibiting caspase activation but also interfering with active caspases. Previous studies in our laboratory have demonstrated that cIAP-1, cIAP-2 and XIAP are expressed in the PC-3 cells [5].

### siRNA mediated knockdown of cIAP1, cIAP2 and XIAP in PC-3 cells

**Figure 2:**
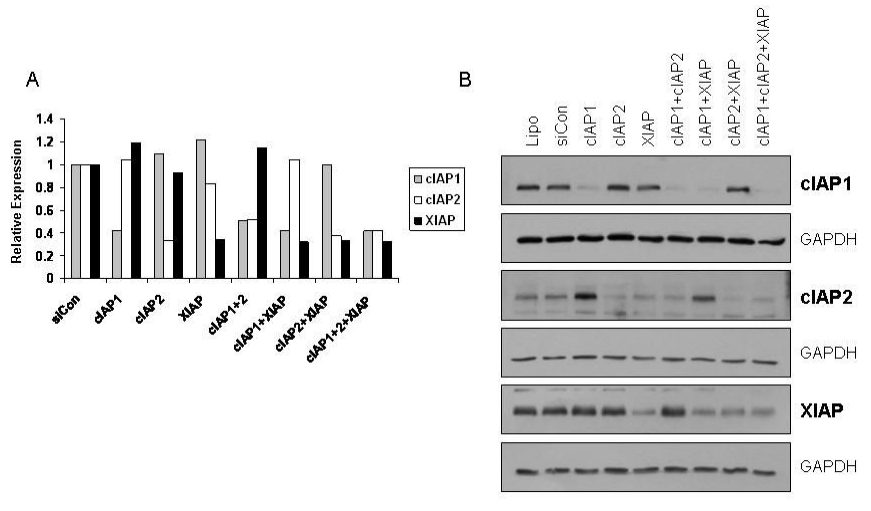
**siRNA mediated knockdown of cIAP1, cIAP2 and XIAP**. Cells were transfected with siRNA targeting cIAP-1 (0.5 nM), cIAP-2 (0.5 nM), XIAP (0.5 nM), or combinations thereof (indicated on x-axis in A, and above top panel in B), and corresponding non-targeting control siRNA (siCon) to a final concentration of 1.5 nM for each group. 24 hour post-transfection, expression levels of targets were analysed at a gene level using real time PCR (A), and at protein level using Western blotting (B). Lipo = lipofectamine control.

In order to determine whether IAP knockdown could increase sensitivity to apoptotic triggers, conditions were optimised using a number of commercially available siRNAs. Optimisation experiments (data not shown) indicated that 0.5 nM siRNA from Ambion was the optimal concentration for efficient knockdown of cIAP-1, cIAP-2 and XIAP in these cells, achieving 60 – 70% reduction of the target gene (Figure 2a). Furthermore, various combinations of siRNAs to different targets did not lead to any decrease in knockdown efficiency for each individual target. Knockdown of IAPs either individually or in combination was also determined at the level of protein by western blotting (Figure 2b) which demonstrated significant reduction in the specific targeted protein.

### Combined knockdown is required to sensitise PC-3 cells to TRAIL induced cell death

**Figure 3:**
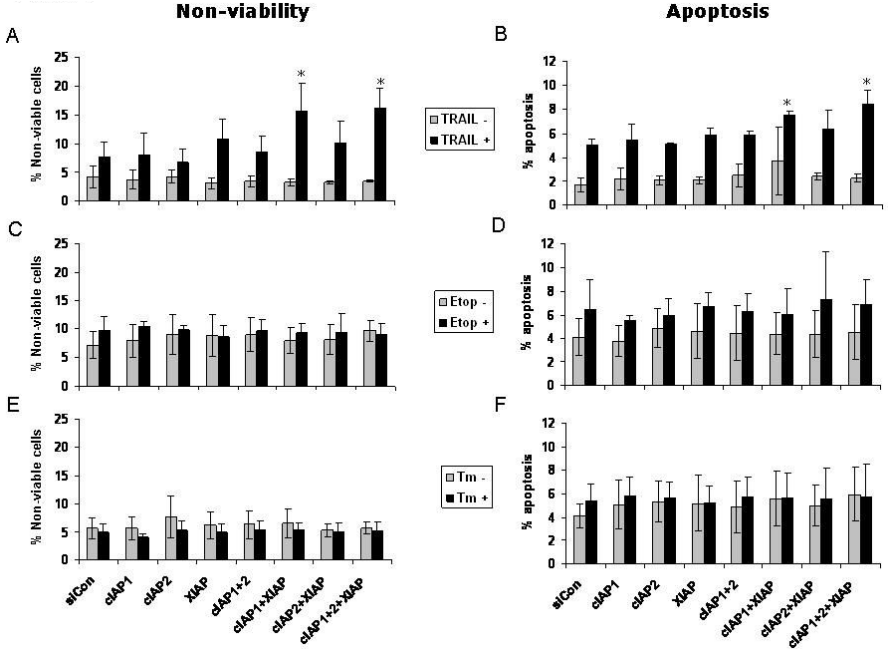
**Combined IAP knockdown is required to sensitise PC-3 cells to TRAIL-induced death**. Cells were transfected with siRNA targeting cIAP-1 (0.5 nM), cIAP-2 (0.5 nM), XIAP (0.5 nM), or combinations thereof (as indicated on x-axis), and corresponding non-targeting control siRNA (siCon) to a final concentration of 1.5 nM for each group. 24 hour post-transfection cells were treated for a further 24 hours with TRAIL (25 ng/ml, A and B), Etoposide (10 μM, C and D), or Tunicmycin (2 μg/ml, E and F). Viability (A, C, E) and Apoptosis (B, D, F) were assessed by propidium iodide DNA staining and subsequent flow cytometric analysis. Values are expressed as mean +/- SD. *Significantly different from cells transfected with non-targeting control (siCon) followed by treatment (TRAIL/Etop/Tm) at p < 0.01.

Following IAP knockdown, cells were treated with TRAIL for 24 hours and cell viability and apoptosis were assessed by flow cytometry. IAP knockdown alone had no effect on cell viability or apoptosis levels as compared with cells transfected with control siRNA (Figure 3). Upon TRAIL treatment however, increased levels of cell death were observed in response to IAP knockdown. Specifically, only combinations of either cIAP-1 and XIAP siRNA, or siRNA to all three IAPs led to a significant increase in cytotoxicity, demonstrated by a small but significant increase in apoptosis levels (Figure 3B), and a drop in viability by up to 20% (Figure 3A). Of note, although there was a trend towards an increase in non-viable and apoptotic cells following knockdown of XIAP alone this did not reach significance. Thus individual knockdown of IAPs does not sensitise PC-3 cells to TRAIL, but combined knockdown is required.

To determine if the effect of IAP knockdown was specific for TRAIL treatment, siRNA mediated knockdown of IAPs was followed by treatment with either etoposide or tunicamycin, agents with distinct modes of action to TRAIL. Like TRAIL, individual knockdown had no effect, however, combined knockdown also failed to increase sensitivity (Figure 3C–F), as demonstrated by measuring viability and apoptosis.

### Increased cell death following IAP knockdown is caspase dependent and leads to increased PARP activation

**Figure 4:**
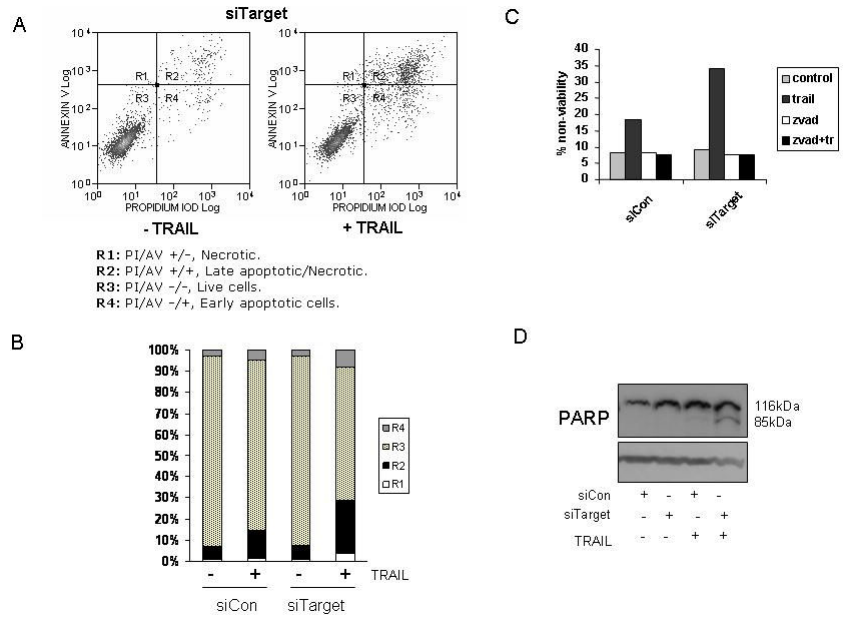
**Increased cell death following IAP knockdown due to apoptosis**. Cells were transfected with the combination of all three siRNAs targeting cIAP-1 (0.5 nM), cIAP-2 (0.5 nM), and XIAP (0.5 nM) (siTarget), or corresponding non-targeting control siRNA (0.5 nM of each control siRNA to a final concentration of 1.5 nM) (siCon). 24 hour post-transfection cells were treated with TRAIL (25 ng/ml) for a further 24 hours. Cell death was assessed by Annexin V/PI DNA staining and flow cytometry. **A**. Representative histograms following combined knockdown of all three IAPs (siTarget) +/- TRAIL treatment. **B**. Percent of cells in each quadrant (R1: PI/AV +/-, Necrotic; R2: PI/AV +/+, Late apoptotic/Necrotic; R3: PI/AV -/-, Live cells; R4: PI/AV -/+, Early apoptotic cells). Representative of three independent experiments. **C**. Cells were pre-treated with zVAD.fmk (100 μM) for 1 hour prior to TRAIL treatment for 24 hours. Viability was assessed by PI-DNA staining and flow cytometry. **D**. Following TRAIL treatment for 9 hours, cell lysates were analysed for PARP processing by Western blot. Western shown is a representative of three independent western blots.

As knockdown was causing a significant loss in viability but only a small detectable increase in apoptosis at 24 hours, we next wanted to determine whether the observed loss in viability was in fact late apoptosis. Initially cell death was assessed by combining Annexin V with PI staining, followed by flow cytometry. As shown in Figure 4A and 4B, this confirmed that combined knockdown of cIAP-1, cIAP-2 and XIAP led to a significant increase in cell death (PI/Annexin V +/+). While these cells are likely late apoptosis, the possibility remained that they are necrotic. To confirm that they were late-apoptosis, cells were incubated with the pan-caspase inhibitor zVAD.fmk prior to TRAIL treatment (Figure 4C). Inhibition of caspase activity using zVAD.fmk completely inhibited the loss in viability induced by TRAIL, thus demonstrating that increased TRAIL induced apoptosis following combined knockdown of IAPs, requires caspase activity. Furthermore, increased processing of the caspase substrate PARP in response to TRAIL, was only observed in cells following triple IAP knock down (Figure 4D).

### IAP knockdown inhibits cell proliferation and clonogenic survival

IAPs have a number of additional pro-survival functions that do not rely on direct apoptosis inhibition. cIAP1 has been implicated as an oncogene [30] and the knock of IAP's including Livin [31] and Survivin [32] inhibit proliferation.

**Figure 5:**
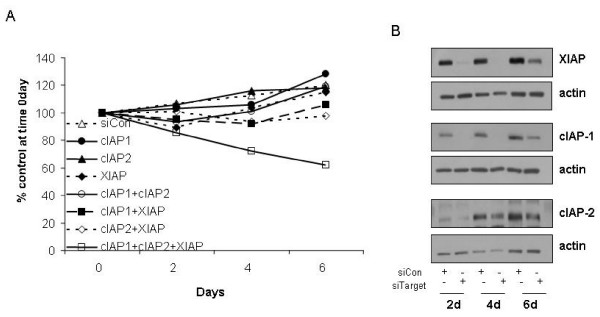
**IAP knockdown inhibits proliferation**. **A**. Cells were transfected with siRNA targeting cIAP-1 (0.5 nM), cIAP-2 (0.5 nM), XIAP (0.5 nM), or combinations thereof (as indicated in insert), and corresponding non-targeting control siRNA (siCon) to a final concentration of 1.5 nM for each group. Cells were grown in 6-well plates from 2 – 6 days, and stained with crystal violet as described in methods. Cell number is expressed as percent of control at time 0 days. Results are a representative of three independent experiments. **B**. Cells were transfected with the combination of all three siRNAs targeting cIAP-1 (0.5 nM), cIAP-2 (0.5 nM), and XIAP (0.5 nM) (siTarget), or corresponding non-targeting control siRNA (0.5 nM of each control siRNA to a final concentration of 1.5 nM) (siCon). Total cellular protein was extracted at days 2, 4 and 6 and western blot analysis of IAP knockdown was undertaken. Western blots are a representative of three individual experiments.

In this context we next wanted to determine if combined IAP knockdown could alter long-term proliferation and clonogenic survival of PC-3 cells. Cells were transfected with siRNAs targeting the IAPs and levels of proliferation were assessed using a crystal violet assay at 2, 3 and 5 day intervals. Figure 5A demonstrates that combined knockdown of IAPs in the absence of any further treatment is sufficient to cause a consistent decrease in cell proliferation over the time-course examined, with almost 40% decrease in proliferation compared with control at day 5 (Figure 5A). This effect was only seen on knockdown of all three IAP in combination. Individual knockdown or knockdown of two IAP in combination had no significant effect on proliferation as demonstrated in Figure 5A which is a representative of three separate experiments. Western blot analysis was also carried out to assess the status of gene knockdown over the course of a week. As demonstrated in figure 5B, significant gene knockdown is maintained for 4 – 6 days. By day six, expression of IAPs started to recover (Figure 5B). Thus transfection with siRNAs leads to efficient knockdown of IAPs for up to a week and corresponds to significant inhibition of cell proliferation.

**Figure 6:**
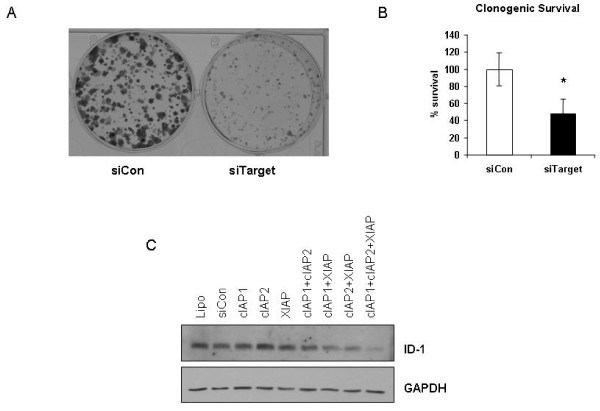
**Short-term knockdown of IAPs decreases long-term survival**. Cells were transfected with the combination of all three siRNAs targeting cIAP-1 (0.5 nM), cIAP-2 (0.5 nM), and XIAP (0.5 nM) (siTarget), or corresponding non-targeting control siRNA (0.5 nM of each control siRNA to a final concentration of 1.5 nM) (siCon). A, B. 24 hour post-transfection cells were re-seeded for clonogenic assay as described in methods. Cells were grown until colonies formed (2 weeks), then stained, fixed and counted to determine surviving fraction. **A**. Representative image of colony formation in Control cells (non-targeting siRNA; siCon) and following combined IAP knockdown (siTarget). **B**. Clonogenic survival in IAP targeted cells. Surviving fraction was calculated as described in methods and expressed as % survival. *p = 0.024 verses siRNA control (siCon), n = 4 individual experiments. **C**. Expression of proliferation marker ID-1 following IAP knockdown as shown by Western blotting.

As short-term knockdown of IAPs had a significant effect on cell proliferation during the duration of knockdown, we wished to investigate the impact on long term survival of cells. Twenty four hours post-transfection cells were reseeded in 6-well plates for clonogenic survival assays and allowed to grow for approximately 2 weeks. Colonies were subsequently stained and counted and clonogenic survival was calculated. As shown in figure 6A and 6B, there was a significant decrease in the numbers of colonies as a result of IAP knockdown. Interestingly this effect was only observed with combined knockdown with individual knockdown of the IAPs having little effect on long term survival (data not shown). To further validate this alteration in proliferation we looked at the expression levels of the proliferation marker ID-1 following IAP knockdown. IAP knockdown using siRNA lead to a decrease in the protein expression levels of ID-1 (Figure 6C). Interestingly, individual knockdown of ID-1 in the PC-3 cells had no effect on clonogenic survival or proliferation (data not shown) indicating that triple IAP knockdown may mediate decreased proliferation through a number of mechanisms, and does not act solely through ID-1.

## Discussion

Androgen independent prostate cancer represents a significant clinical problem emerging 2–3 years after the commencement of hormone ablation therapy. There is no known cure for this progressive form of the disease but manipulation of the resistant anti-apoptotic phenotype has been proposed as a treatment strategy. Previous studies have demonstrated that androgen independent prostate cancer cells have elevated levels of a range of anti-apoptotic proteins including the inhibitors of apoptosis family of proteins (IAPs), which is the basis for targeting these proteins in this study. Here we confirm that PC-3 cells have significant expression of cIAP-1, cIAP-2 and XIAP as compared to PwR-1E and LNCaP cells [6,5] and are highly resistant to TRAIL-induced apoptosis, as we have previously demonstrated [29].

We have previously reported that pre-incubation of PC-3 cells with resveratrol significantly increases their sensitivity to TRAIL-induced apoptosis due to an inhibition of AKT phosphorylation associated with altered IAP expression. This study clearly demonstrated that manipulation of the survival signals within the cell can increase therapeutic responses, however targeted approaches and a clear understanding of how they mediate their effects is required. Despite PC-3 cell resistance to TRAIL induced apoptosis we did demonstrate activation of caspase 8 and 3 activity. This is in keeping with recent studies by Hellwig et al who have shown that a required threshold of caspase activity must be reached before apoptosis can be induced [33]. From this we hypothesised that decreasing the expression of the inhibitors of apoptosis would remove the block or lower the threshold required to allow TRAIL to initiate apoptosis in PC-3 cells.

In keeping with this hypothesis we developed siRNA strategies to simultaneously inhibit the expression of cIAP-1, cIAP-2 and XIAP, which were reduced to about 40% of control levels as confirmed at both the mRNA and protein level. This was not associated with any direct toxicity or loss of cell viability within the early time points studied. We clearly demonstrated that individual knockdown of a single IAP had no effect on the expression levels of the other two IAP. This is supported by Volger et al who showed similar results when they individually knocked down XIAP using retroviral short-hairpin RNA vector [34]. In contrast, other studies have shown that XIAP inhibition causes up regulation of cIAP-1 and cIAP-2 [35]. We also demonstrate that IAP knock down does not increase basal apoptosis. Previously we have shown that anti-sense oligonucleotide strategies to cIAP-1 did result in a significant 10% induction of basal apoptosis in PC-3 and DU-145 cells [15]. In addition, knock down of XIAP using antisense did, by itself, result in a small but significant decrease in cell viability in DU-145 cells [21]. We believe that the differences observed in these studies and that of the current study might be as a result of the strategies used to knockdown the individual IAPs and the level of knock down achieved. These other strategies may have effects that are directly toxic to the target cells and thus on combination with other triggers of apoptosis pushes them into a death response.

Following confirmation of appropriate knock down we demonstrate for the first time that triple knockdown of cIAP-1, cIAP-2 and XIAP increases the sensitivity of PC-3 cells to TRAIL induced loss in viability and apoptosis as compared to individual IAP knock down. This demonstrates that the resistance to TRAIL induced apoptosis in PC-3 cells is facilitated by all three IAPs. But, in fact we only partially increased the sensitivity of these cells to apoptosis indicating that other pathways are also involved. When we compare the levels of apoptosis induced by the triple knock down and TRAIL with a combination of Resveratrol and TRAIL, as used in previous studies, the levels of apoptosis are higher with Resveratrol pre-treatment (data not shown). This is in keeping with the ability of resveratrol to not only alter IAP expression but also to affect other survival pathways including AKT signalling [29].

Individual knock down of cIAP-1, cIAP-2 and XIAP did not increase sensitivity to apoptosis despite previous studies demonstrating that the knock down of XIAP can increase sensitivity to TRAIL and cisplatin induced apoptosis [21], and that knock down of cIAP-1 can sensitise for Fas and TNFalpha induced apoptosis [15]. In our current study, the next most effective combination was cIAP-1 and XIAP which resulted in significant induction of apoptosis and loss of viability following treatment with TRAIL.

Targeting IAPs either individually or in combination did not increase sensitivity of the cells to Etoposide or Tunicmycin, treatments which where chosen on the basis that their effects are partially mediated via disruption of mitochondrial or endoplasmic reticulum signalling [29]. This suggests selectivity of this priming effect for the death receptor and extrinsic cell death pathway, and is in keeping with our previous study where cIAP-1 antisense primed only for Fas and TNFalpha induced apoptosis but not for paclitaxel induced apoptosis. In contrast, knockdown of XIAP in DU145 cells has been shown to increase sensitivity to cisplatin induced apoptosis and its knockdown in other tumour cells has been shown to sensitise for cytotoxic drugs such as mitomycin C, doxorubicin, etoposide and taxanes [36]. This suggests not only cell-type dependent effects but also treatment specific effects in relation to individual or combinations of IAPs.

Having demonstrated that triple knock down increases apoptosis and decreases viability in response to TRAIL we next wanted to investigate the mechanisms involved. We demonstrated that this effect is caspase dependent as there is a significant increase in PARP cleavage, and the priming effect could be prevented by pre-incubation with the pan-caspase inhibitor ZVAD.fmk. This suggests that the threshold level of caspase activation required to initiate apoptosis can be reached when cIAP-1, cIAP-2 and XIAP are knocked down, an effect which is blocked by ZVAD.fmk.

IAPs have a number of additional pro-survival functions that do not rely on direct apoptosis inhibition. cIAP-1 has been implicated as an oncogene whereby it promotes tumourogenesis in cooperation with Yap1 [30]. Similarly, there is emerging evidence that knock down of IAPs including Livin and Survivin inhibit proliferation. Down regulation of Livin expression by RNAi in colorectal cancer cell lines significantly inhibited in vitro cell proliferation and in vivo tumorigenicity [31], while knock down of the survivin gene induced growth inhibition, as well as apoptosis, in Burkitt's lymphoma Raji cell line [32]. Others have demonstrated that knockdown of XIAP strongly inhibits clonogenicity of pancreatic cancer cells treated with TRAIL indicating that XIAP promotes clonogenic survival of pancreatic carcinoma cells [34].

Another very important role for the IAP is in their regulation of NF-κB. cIAP-1 acts as an E2 ubiquitin ligase for nuclear factor-κB-inducing kinase (NIK) maintaining low basal levels of NIK and preventing NF-κB signalling [37] thus loss of cIAP-1 could result in NF-κB activation. This contradictory role for cIAP-1 might well explain why IAP knockdown does not sensitise for cytotoxic induced apoptosis with Etoposide. One hypothesis would be that IAP knockdown could result in NF-κB activation and combined with Etoposides stimulation of NF-κB result in increased NF-κB activation, transcription of anti-apoptotic proteins and survival of the cell. On the other hand, where TRAIL has also been shown to induce NF-κB activation as a survival signal this effect is dependent on NIK, IKK1, IKK2 as well as TRAF2 [38]. As cIAP-1 binds to TRAF2 it might prevent TRAIL induced NF-κB activation resulting in increased susceptibility to apoptotic triggers and begin to explain the specific effect of IAP knockdown on TRAIL induced apoptosis.

In our current study, combined knockdown of cIAP-1, cIAP-2 and XIAP was associated with a decreased rate of proliferation as assessed by crystal violet growth assays. More interesting, long term clonogenic assays also demonstrated reduced colony formation after 2 weeks following a transient knock down of cIAP-1, cIAP-2 and XIAP. This study clearly demonstrates that manipulation of these three IAP simultaneously has a direct effect on the cells ability to proliferate and recover. To further understand the mechanism involved we investigated the effects of the triple knock down on ID-1 expression. ID-1 is a member of the helix-loop-helix protein family and regulates gene transcription with significant increases in activity in actively proliferating cells [39]. Associated with the activation of NF-κB as a down stream target, it mediates a number of functions from proliferation, survival, angiogenesis and invasion leading to tumorigenesis, and represents an important oncogene [39]. Here ID-1 protein expression was decreased following 24 hour simultaneous knock down of cIAP-1, cIAP-2 and XIAP, with no effect of individual IAP manipulation on its expression.

## Conclusion

This study demonstrates that simultaneous knock down of cIAP-1, cIAP-2 and XIAP primes PC-3 cells for TRAIL induced apoptosis, an effect which is caspase dependent. The inability to alter sensitivity to other triggers of apoptosis suggests that this effect is specific for death receptor pathways. Thus IAP knock down might facilitate the immune-surveillance mechanisms to counter cancer progression and, in combination with TRAIL or similar therapeutics, could represent an important treatment strategy. We have also shown that triple knock down has a direct effect on cell colony formation and proliferation indicating a central role of the IAP in this function and suggesting the potential benefits of targeting IAPs even in the absence of other treatments.

## Competing interests

This work was supported by Cancer Research Ireland CRI03WAT. The authors declare that they have no competing interests.

## Authors' contributions

CG carried out the design of the siRNA, knockdown experiments and functional assessment of apoptosis viability and proliferation and participated in the design of the study and analysis of the results. CD undertook the clonogenic assays and design of the IAP siRNA. AON undertook the ID-1 experiments and analysis of the results. RWW participated in the development of the central concept and design of the study and analysis of the results. All authors read and approved the paper.
